# Enhanced secondary pollution offset reduction of primary emissions during COVID-19 lockdown in China

**DOI:** 10.1093/nsr/nwaa137

**Published:** 2020-06-18

**Authors:** Xin Huang, Aijun Ding, Jian Gao, Bo Zheng, Derong Zhou, Ximeng Qi, Rong Tang, Jiaping Wang, Chuanhua Ren, Wei Nie, Xuguang Chi, Zheng Xu, Liangduo Chen, Yuanyuan Li, Fei Che, Nini Pang, Haikun Wang, Dan Tong, Wei Qin, Wei Cheng, Weijing Liu, Qingyan Fu, Baoxian Liu, Fahe Chai, Steven J Davis, Qiang Zhang, Kebin He

**Affiliations:** School of Atmospheric Sciences, Nanjing University, Nanjing 210023, China; School of Atmospheric Sciences, Nanjing University, Nanjing 210023, China; Chinese Research Academy of Environmental Sciences, Beijing 100012, China; Department of Earth System Science, Tsinghua University, Beijing 100084, China; State Key Joint Laboratory of Environment Simulation and Pollution Control, School of Environment, Tsinghua University, Beijing 100084, China; School of Atmospheric Sciences, Nanjing University, Nanjing 210023, China; School of Atmospheric Sciences, Nanjing University, Nanjing 210023, China; School of Atmospheric Sciences, Nanjing University, Nanjing 210023, China; School of Atmospheric Sciences, Nanjing University, Nanjing 210023, China; School of Atmospheric Sciences, Nanjing University, Nanjing 210023, China; School of Atmospheric Sciences, Nanjing University, Nanjing 210023, China; School of Atmospheric Sciences, Nanjing University, Nanjing 210023, China; School of Atmospheric Sciences, Nanjing University, Nanjing 210023, China; School of Atmospheric Sciences, Nanjing University, Nanjing 210023, China; School of Atmospheric Sciences, Nanjing University, Nanjing 210023, China; Chinese Research Academy of Environmental Sciences, Beijing 100012, China; Chinese Research Academy of Environmental Sciences, Beijing 100012, China; School of Atmospheric Sciences, Nanjing University, Nanjing 210023, China; Department of Earth System Science, Tsinghua University, Beijing 100084, China; Department of Earth System Science, University of California, Irvine, CA 92697, USA; Jiangsu Environmental Monitoring Center, Nanjing 210036, China; Jiangsu Environmental Monitoring Center, Nanjing 210036, China; Jiangsu Provincial Academy of Environment Science, Nanjing 210036, China; Shanghai Environmental Monitoring Center, Shanghai 200030, China; Beijing Key Laboratory of Airborne Particulate Matter Monitoring Technology, Beijing Municipal Environmental Monitoring Center, Beijing 100048, China; Chinese Research Academy of Environmental Sciences, Beijing 100012, China; Department of Earth System Science, Tsinghua University, Beijing 100084, China; Department of Earth System Science, University of California, Irvine, CA 92697, USA; Department of Earth System Science, Tsinghua University, Beijing 100084, China; Department of Earth System Science, Tsinghua University, Beijing 100084, China; State Key Joint Laboratory of Environment Simulation and Pollution Control, School of Environment, Tsinghua University, Beijing 100084, China

**Keywords:** COVID-19, haze pollution, ozone, emission reduction, secondary pollution

## Abstract

To control the spread of the 2019 novel coronavirus (COVID-19), China imposed nationwide restrictions on the movement of its population (lockdown) after the Chinese New Year of 2020, leading to large reductions in economic activities and associated emissions. Despite such large decreases in primary pollution, there were nonetheless several periods of heavy haze pollution in eastern China, raising questions about the well-established relationship between human activities and air quality. Here, using comprehensive measurements and modeling, we show that the haze during the COVID lockdown was driven by enhancements of secondary pollution. In particular, large decreases in NO_x_ emissions from transportation increased ozone and nighttime NO_3_ radical formation, and these increases in atmospheric oxidizing capacity in turn facilitated the formation of secondary particulate matter. Our results, afforded by the tragic natural experiment of the COVID-19 pandemic, indicate that haze mitigation depends upon a coordinated and balanced strategy for controlling multiple pollutants.

## INTRODUCTION

Efforts to control the spread of the 2019 novel coronavirus (COVID-19) have drastically reduced human activities worldwide [1,2]. As one of the epicenters of the pandemic, China was the first country to shut down commercial activities, restrict travel and require its people to stay home beginning in late January 2020 [2–4]. These restrictions are believed to have drastically decreased air pollutant emissions. For example, the TROPOMI instrument on the Sentinel 5P satellite observed approximately 65% decrease in tropospheric NO_2_ columns over the eastern China region compared to the same period in 2019 [3,5,6]. Previous studies have shown that heavy haze pollution in eastern China has primarily been driven by accumulated anthropogenic emissions together with rapid secondary production [7–13]. Indeed, strict policies reduced anthropogenic air pollution emissions between 2013–17 and have been the main driver of decreases in PM_2.5_ (particles with an aerodynamic diameter smaller than 2.5 μm) pollution in China [13–16].

Yet despite large reductions in primary pollutant emissions, there were several heavy haze events over eastern China during the COVID-19 lockdown, which have seeded doubt among the Chinese public and policymakers regarding the current scientific understanding of the mechanisms of haze pollution. Here, using comprehensive measurements of the mass and chemical compositions of PM_2.5_ as well as related trace gases, together with numerical model simulations, we show that secondary pollution during the COVID lockdown was unexpectedly enhanced as the result of imbalances in the reduction of primary emissions. Our findings thus underscore the importance of a carefully tailored and balanced strategy of emission control to reduce haze pollution in China.

## OBSERVATIONAL EVIDENCES OF ENHANCED SECONDARY PM FORMATION

**Figure 1.**
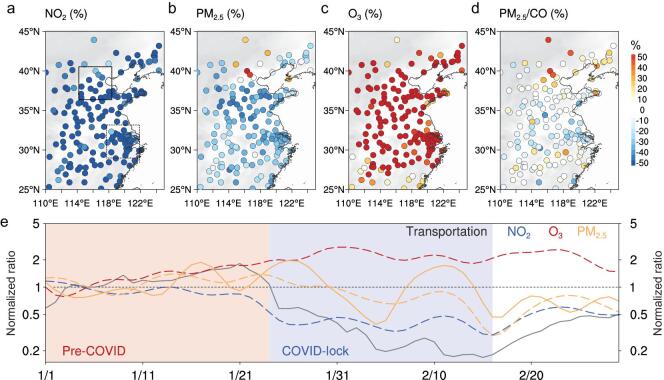
Changes in air quality before and during the COVID-19 lockdown in eastern China. **a–d,** Differences in averaged concentrations of NO_2_, PM_2.5_, O_3_ and PM_2.5_/CO ratio before (1–23 January 2020) and during (26 January–17 February 2020) the COVID-19 lockdown periods at air quality monitoring stations in eastern China. The dashed and solid rectangles in **a** represent YRD and BTH region, respectively. **e,** Time series of normalized change in NO_2_, O_3_, PM_2.5_ in eastern China (30°N–40°N, 110°E–120°E) and transportation index in China in January–February 2020. Note: the normalized ratio is calculated using time series with daily variation removed by EEMD filter and scaled with the first 10-day average. The dashed and solid yellow lines in **e** represent the averaged PM_2.5_ concentrations in YRD and BTH cities, respectively.

Figure 1 and Fig. S1 in the Supplementary Data show the large change in pollutant emissions during the three weeks of the Chinese New Year holiday and the COVID lockdown that immediately followed (the lock period) in comparison with the three weeks before the Chinese New Year (pre-period), based on the real-time measurement by air quality monitoring network. Nitrogen dioxide (NO_2_) levels decline sharply (by >60%) during the lockdown, consistent with both the reduction in human activities reflected by the transportation index (Fig. 1a and e) and satellite observations (Fig. S2) [3,5,6]. Other primary gaseous pollutants such as carbon monoxide (CO) and sulfur dioxide (SO_2_) show patterns similar to NO_2_ (Fig. S1). Yet concentrations of PM_2.5_—perhaps the most important air pollutant from a public health perspective—do not show the same decrease over eastern China, but instead increase in the area of Beijing, Tianjin and Hebei (BTH) and in the northern part of China (Fig. 1b and e). Meanwhile, ozone (O_3_), which is an important secondary pollutant in warmer months but generally of less concern in winter [17–19], was also substantially enhanced (in some cases by >100%) over all of eastern China (Fig. 1e) [6]. The distribution of pollutant levels shows systematic shifts in O_3_ and NO_2_ in the heavily populated BTH and Yangtze River Delta (YRD) areas of eastern China during the COVID lockdown, with less change in PM_2.5_, particularly in the BTH region (Fig. S3). The ratio of PM_2.5_/CO, an indicator of secondary pollutants to primary emission, reveals a large-scale enhancement throughout northern China (Fig. 1d). The anomalies in all these species diminished near the end of February, when COVID restrictions began to be eased and people outside of the Hubei province started back to work (Fig. 1e) [2].

The observed changes in primary pollutants are proportionate to reductions in emissions during the COVID lockdown. Emissions estimates based on up-to-date activity levels suggest an overall reduction of NO_x_ about 60%–70% in eastern China, 70%–80% of which was related to road traffic and 20%–25% from industry and power plants (Table S1). Model simulations with fixed emission inventory without reduction considered (to exclude the possible influence of synoptic weather on air pollution) [4,20,21] show very large bias from observations, which also confirms the change in primary reduction (NO_x_) and elevated O_3_ and PM_2.5_, especially in the BTH region (Fig. S4). Importantly, studies of previous changes in pollution emissions have never found such a pronounced increase in secondary pollutants like O_3_ and PM_2.5_ [22–25]. But the reduction in primary pollutants during the COVID lockdown is also more extreme than those observed previously (e.g. during past holidays or special events such as the 2008 Olympics or the 2014 meeting of the Asia-Pacific Economic Cooperation in Beijing) [22,26,27].

**Figure 2.**
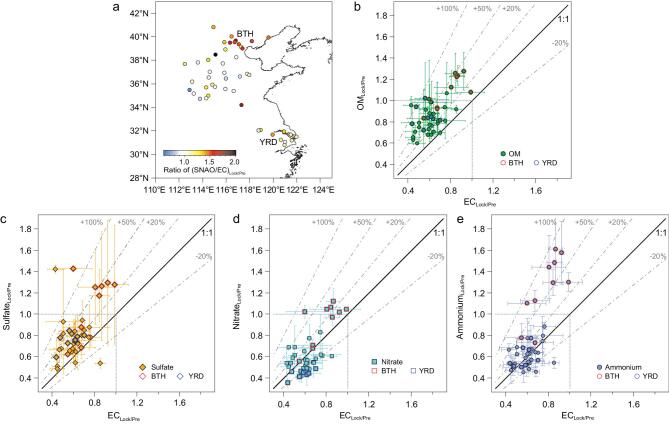
Observational evidences of enhanced secondary PM production in eastern China during the COVID-19 period. **a,** Spatial distribution of the ratio of total secondary PM (sulfate, nitrate, ammonium and organic matters) versus element carbon (EC), i.e. (SNAO/EC)_Lock/Pre_**,** between the COVID-lock and pre-COVID periods. **b–e,** Scatter plots of the ratios of organic matters, sulfate, nitrate and ammonium during COVID-lock versus pre-COVID periods as a function of the ratio of EC during the two periods. Error bars present the standard deviations. Data of cities in the BTH and YRD regions are marked in red and blue edge line, respectively.

Previous studies have suggested many different processes of secondary PM formation in northern China [7,8,10–12,28]. To investigate the causes for the increase in PM_2.5_ in eastern China during the COVID lockdown, we examine the chemical compositions of PM_2.5_ from a regional network in northern and eastern China. Because elemental carbon (EC) is mainly from primary emissions [29], the relative change in concentration of secondary species, e.g. sulfate, nitrate, ammonium and organic matter (SNAO), provides information on the enhancement of secondary PM formation. Given that secondary organic aerosol (SOA) has been reported to dominate the OM (organic matters) in megacities in China [8] and field measurements in both Beijing and Nanjing during the study period is also indicative of the dominance of SOA in the OM (Fig. S5), here the relative change of OM to EC could reveal the secondary OM production. As shown in Fig. 2a, secondary PM production during the lockdown was especially high in eastern China in comparison to the pre-period, especially in the BTH region (with a ratio of ΔSNAO/ΔEC as high as 1.5). Such secondary formation is also consistent with the PM_2.5_/CO ratios from the air quality monitoring network (Fig. 1d).

The scatter plots of the ΔSNAO/ΔEC during the lockdown indicate an overall reduction in EC for all stations (the ratio of EC_Lock/Pre_ lower than 1 with a minimum as low as 0.4), but a substantial enhancement in secondary PM formation, shown by the much higher ratio of ΔSNAO/ΔEC_Lock/Pre_ (Fig. 2b–e). In these scatter plots, the dots above the 1:1 line generally indicate higher proportion and an enhanced secondary production rate, but those with the y-axis above 1.0 mean increased concentrations, i.e. the reduction of primary emission offset by secondary products. For OM, sulfate and ammonium, the ratio is particularly high, ∼20%–100% (Fig. 2b, c and e), but for nitrate, the ratio is relatively low (∼−30%–60%), with some cities in the YRD and Henan Province below the 1:1 line (Fig. 2d), indicating weakened nitrate formation associated with the overall changes in NO_2_ and O_3_ [9]. The decreased nitrate formation in regions other than the BTH could explain why the PM_2.5_/CO ratio mainly increased in northern China (Fig. 1d). Although the difference in reduction rate of different primary OM sources may also affect the OM/EC_Lock/Pre_ ratio, given the high portion of secondary organics in megacities in China [8], the significantly high ratio in Fig. 2b indicates a substantial influence from enhanced production of secondary OM. Available field measurements in Beijing and Nanjing also confirmed a substantial stronger enhancement of secondary OM than primary (Fig. S5). However, for the BTH region all secondary inorganic aerosols, e.g. sulfate, nitrate, ammonium, and organics show significantly higher net chemical production (see the ratio of SNAO_Lock/Pre_ over 1.0, marked in red), thereby a pretty high concentration of PM_2.5_ in northern China, as shown in Fig. 1.

The diurnal cycle of the ΔSNAO/ΔEC_Lock/Pre_ ratio further reveals the main processes that influence secondary PM. As shown in Fig. S6c, the secondary production of all PM species in the BTH area were enhanced (positive values) most of the day during the lockdown period, with the enhancement extending from midnight to around 9:00 LT (local time) in the morning, indicating an important role of nighttime chemistry. The enhancement of secondary aerosols coincides with the rise in ozone (over 100%) and also O_3_^*^NO_2_, a proxy of  NO_3_ radical, which is a vital oxidant for nighttime secondary PM formation [30–34]. However, in other regions, such as YRD, the diurnal cycle of these ratios shows an overall positive value, except for nitrate, which was consistent with the change of O_3_^*^NO_2_ proxy (Fig. S6d). These results imply that—although secondary haze formation was generally enhanced during the COVID lockdown—the magnitude of such secondary PM production was different in different regions, depending on O_3_ and the NO_3_ radical. This suggests the great importance of NO_x_ on both daytime and nighttime atmospheric chemistry [31,35–38].

## MODEL SIMULATIONS AND UNDERSTANDING OF THE DOMINANT MECHANISM

**Figure 3.**
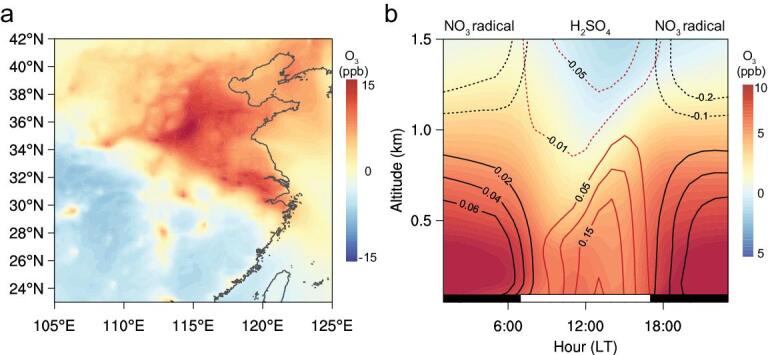
Modeling evidences of enhanced atmospheric oxidizing capacity in eastern China due to the COVID-19 lockdown. **a,** Spatial distribution of averaged near-surface ozone enhancement by emission reduction due to the COVID-19 lockdown. **b,** Diurnal cycle of averaged vertical distribution of O_3_, NO_3_ radical and sulfuric acid in eastern China (30°N–40°N, 110°E–120°E). The black lines mean the contour of NO_3_ radical (unit: ppt), and the red lines represent that of sulfuric acid (unit: ppt). The negative values are shown using dashed lines. The black and white bar near the x axis in **b** indicates the nighttime and daytime of a day.

We further conduct model simulations using the Weather Research and Forecasting model coupled with Chemistry (WRF-Chem), based on an up-to-date emissions inventory estimated by dynamically adjusted human activity levels (Table S1 and Fig. S7). As aforementioned, we find significant decreases in atmospheric NO_x_, due mainly to lower vehicle emissions, which could cause a substantial increase in the availability of O_3_ and nighttime NO_3_ radical in eastern China (Fig. 3). Moreover, the simulations reproduce the enhancements of both O_3_ and NO_3_ radical in eastern China, a region with intensive anthropogenic emissions of NO_x_ [39,40], and a decrease in southern and southwestern China (Figs 3a and S8). The diurnal cycles from both model simulations (Fig. 3b) and observation-based diagnose (Fig. S6) show that the relative changes in O_3_ and NO_3_ radical concentrations during the pre/lock periods are significant, particularly during nighttime, because of strong NO-titration existed in the pre-lockdown period (Figs 3b and S8). Accordingly, the NO_3_ radical increased a lot below the altitude of 1 km during the night, even if its precursor NO_x_ declined sharply.

The enhanced atmospheric oxidizing capacity accelerated the formation of sulfuric acid (H_2_SO_4_) in the boundary layer [41,42]. Comparatively, this kind of enhancement is much stronger in the BTH region than the YRD region. By diagnosing the changes in HNO_3_ and nitrate in WRF-Chem simulations (Fig. S9), we can explain the differences in diurnal cycle of nitrate changes in BTH and other regions (Fig. S6). Here we mainly examine those gaseous intermediate products directly linked with oxidants and secondary PM formation averaged over a regional scale. In the BTH region, a substantial increase of HNO_3_ occurs in the boundary layer almost the entire day with a positive nitrate formation occurring from night to early morning in the lower PBL (planetary boundary layer). However, in the YRD region, HNO_3_ and nitrate show an overall decrease during the COVID lockdown (Fig. S9), consistent with observations (Figs 2c and S6d).

As a result of the enhanced oxidizing capacity, the model shows a significant increase in sulfuric acid production during the daytime in eastern China (Figs 3 and S8). Observations at the Station for Observing Regional Processes of the Earth System (SORPES) in Nanjing also show an increase in sulfuric acid concentration (∼30% increase) in both the day and night of sunny days (Fig. S10). Consequently, vigorous new particle formation processes and stronger sulfate production were frequently observed at the station during the COVID lockdown. It is also worth noting that a substantially higher OM fraction at night during the lockdown period was associated with stronger daytime new particle formation (NPF) events (Fig. S10), implying that the enhanced formation of secondary PM was due to a stronger nighttime oxidization by both O_3_ and NO_3_ radical [31,32,43,44], even under the condition of decreasing precursors (i.e. volatile organic compounds (VOCs), SO_2_ and NO_x_).

**Figure 4.**
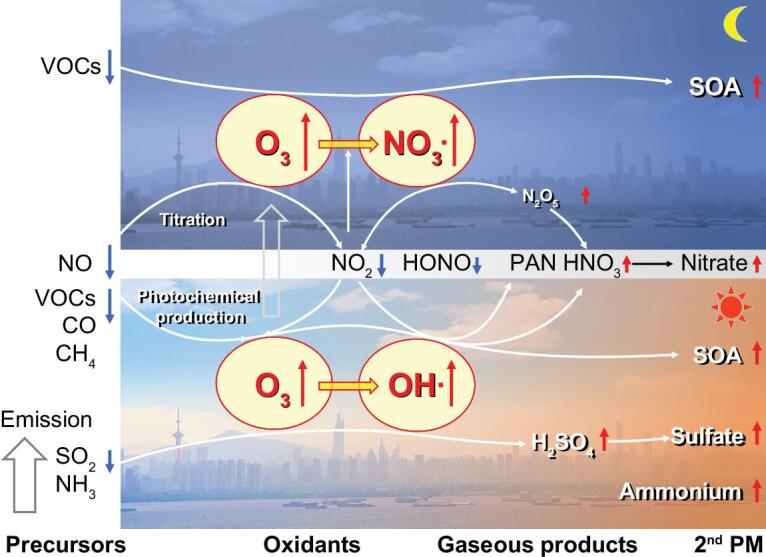
Conceptual model showing how the secondary formation offsets the reduction of primary pollutions in China with different processes during the day and night. Note: the upper and lower panels, indicated by moon and sun, represent nighttime and daytime processes, respectively. Blue downward arrows mean the reduction of emission or decrease of concentrations in the atmosphere, while the red upward arrows mean enhancement of production in the atmosphere. The length of these arrows indicates the degree of change. SOA, secondary organic aerosol; PAN, peroxyacetyl nitrate; HONO, nitrous acid.

Figure 4 illustrates such non-linear relationship of reduced precursors causing enhanced oxidants and secondary products over a regional scale. The elevated oxidizing capacity will enhance the proportion of secondary PM in regional-scale haze. Under typical meteorological conditions, such enhancement of secondary PM formation could cause even higher concentrations and hence offset the efforts of a substantial reduction in primary emissions in sub-regional scale, like the BTH (Fig. S11). The non-linear relationship between NO_x_ reduction and oxidant enhancement is also evident in measurements from air quality monitoring networks. As shown in Fig. S12, the relationship of O_3_ and night O_3_^*^NO_2_ proxy vs. NO_2_ in BTH and YRD regions shows distinct patterns during the COVID-lock and pre-COVID periods. A more substantial enhancement of oxidizing capacity, for both O_3_ and NO_3_ radical, took place in the BTH region.

**Figure 5.**
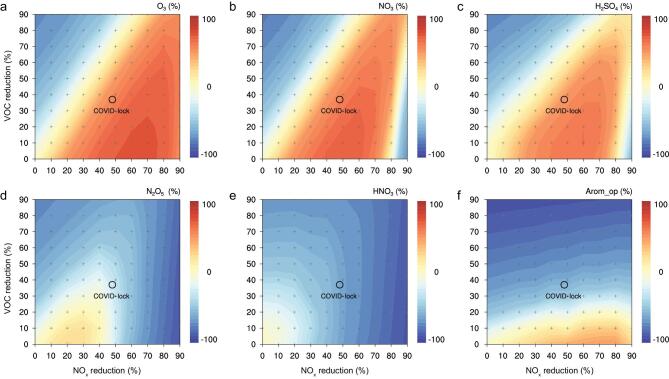
Response of atmospheric oxidizing capacity and gaseous oxidation products to synergetic emission reduction of VOCs and NO_x_ in eastern China. **a–f,** EKMA isopleths of relative changes of O_3_, NO_3_ radical, H_2_SO_4_, N_2_O_5_, HNO_3_ and Arom_op as a function of VOCs and NO_x_ emission reduction in eastern China during the COVID-19 lockdown period. Note: eastern China is defined as the domains of 30°N–40°N, 110°E–120°E. Arom_op means oxidization products of aromatics. The open circles marked in the figures indicate the averaged emission reduction rate of VOCs and NO_x_ during the COVID-19 lockdown period. The crosses indicate the matrix of the simulation scenarios to generate these contour plots.

By testing the sensitivity of our WRF-Chem simulations to different NO_x_ reduction rates (10%–90%), we find a non-linear response in both O_3_ and NO_3_ radical in eastern China (Fig. S13). These results show that the sudden and precipitous reduction in NO_x_ emissions could cause a substantial increase of O_3_, NO_3_ radical and hence a non-linear response of oxidation products like H_2_SO_4_, HNO_3_ and N_2_O_5_ and oxygen-containing organic compound, which have a direct linkage to the enhancement of secondary PM species. In the BTH region, the enhancement is extremely significant because of the much higher NO_x_ concentrations and weak incident solar radiation in northern China, which makes the NO-titration and O_3_-VOCs-NO_x_ photochemistry more sensitive to the NO_x_ reduction [37]. Figure S13 also suggests that a further reduction of NO_x_ (e.g. to 60%–70% in BTH and 50%–60% in YRD, achieved from industrial or residuals sectors [45]) would reach a tipping point for decreasing oxidizing capacity. Unfortunately, the emission reductions in both regions during the COVID lockdown were almost at the peak for secondary production. A concurrent reduction in VOCs could also have reduced the non-linear relationship. As shown in Fig. S14, synchronous VOCs reduction could have partly counterbalanced the enhancement of secondary pollution due to such non-linear response. Based on matrix-type WRF-Chem simulations considering the synergetic effects from NO_x_ and VOCs emission reduction, we obtained EKMA (empirical kinetic modeling approach) isopleths for main oxidants and gaseous oxidation products for eastern China (Fig. 5). They clearly demonstrate the high non-linear dependence of oxidants and secondary PM formation upon the reduction of primary emissions.

## CONCLUSION AND POLICY IMPLICATIONS

Our results show that the dramatic reductions in NO_x_ and other air pollution emissions during China's COVID lockdown led to substantial increases in O_3_, which in turn increased atmospheric oxidizing capacity and enhanced formation of secondary PM in eastern China. In China, winter haze and summer ozone pollution are the two major air quality challenges, but with different and separated control policies. To mitigate haze pollution, policies have focused on the reduction of primary emissions such as SO_2_, NO_x_ and NH_3_ [14,46,47], while efforts have been proposed to reduce ozone pollution target VOCs [18]. Although some works [48–50] partly investigated the impact of primary emission to secondary pollution based mainly on modeling in different regions, based on the natural passive control experiment during the COVID lockdown, here our study shows direct and consistent ‘observational’ and modeling evidences on the non-linear relationship of emission reduction and secondary haze pollution in the real world. Regulation of SO_2_ and NO_x_ emissions imposed since 2013 have successfully reduced haze pollution in eastern China [14,15], while our results suggest that the benefit of proposed further reductions in primary emissions [45,51] might be offset by enhanced secondary formation of PM. Thus, the lockdown imposed to protect public health during the COVID pandemic has shown that efforts to further decrease PM_2.5_ pollution in eastern China may be more challenging than anticipated. Specifically, a non-linear tipping point of NO_x_ chemistry will require that future reductions in China's haze pollution manage the balance of emitted species, with different consideration of VOCs and NO_x_ ratios among different regions.

## MATERIALS AND METHODS

### Observational data and analysis method

Concentrations of NO_2_, O_3_, CO, SO_2_, PM_2.5_ and PM_10_ at more than 1500 stations are archived at the air monitoring data center of Ministry of Ecology and Environment of China. To demonstrate pollution variations, ensemble empirical mode decomposition (EEMD) is applied to decompose observational data. In addition, PM_2.5_ chemical compositions are recorded in more than 40 cities. In Nanjing, we also conducted observations on particle size distribution, VOCs and other trace gases at the SORPES station. TROPOspheric Monitoring Instrument (TROPOMI) provides retrievals of NO_2_ column amount [52], which are also employed to illustrate the spatial pattern and temporal variation of air pollution around COVID-19 in China. Detailed information on observational data can be found in the supplementary text.

### Emission reduction estimation due to the lockdown control

Emission reduction is estimated using the bottom-up inventory model of  Multi-resolution Emission Inventory for China (MEIC), developed by Tsinghua University [51]. To estimate emission reductions due to COVID-19 lockdown, we update China's emissions data to January and February 2020 based on dynamic economic and industrial activity levels. Descriptions on emission reduction estimation due to the lockdown control are detailed in the supplementary text. The estimation of provincial emission reduction ratio of main trace gases and primary PM is presented in Table S1.

### Regional air quality modeling

Coupled dynamical and chemical simulations are conducted based on WRF-Chem model. This model is demonstrated to be able to reproduce pollution in China and the configurations are given in detail in previous works [53,54]. The base simulation was conducted from 1 December 2019 to 5 March 2020 by using MEIC emission inventory. We then use the up-to-date reduction ratio to understand the emission-triggered perturbations. Furthermore, hundreds of parallel simulations with various NO_x_ and VOCs emission scenarios were performed to investigate the relationship between primary emission and secondary pollution. Given that current air quality models still face challenges for accurately characterizing secondary PM and we mainly focus on the enhancement of secondary PM formation, it is feasible to reveal the sensitivity of secondary PM based on their precursors like H_2_SO_4_, HNO_3_ and VOC oxidation product. Furthermore, the Lagrangian particle dispersion model HYSPLIT was used to identify transport pathways and to track potential sources. Global Data Assimilation System (GDAS) data was used to drive the model and 3000 particles released every hour from the location of concern were then tracked backward for 7 days to identify ‘footprint’ retroplume [15].

## DATA AVAILABILITY

Daily satellite retrievals of NO_2_ column amount are openly accessible at http://www.temis.nl/airpollution/no2col/data/tropomi. The daily transportation index is provided by Baidu migration dataset (https://qianxi.baidu.com). NCEP (National Centers for Environmental Prediction) FNL (Final) and ADP (Automated Data Processing) observational data can be obtained from https://rda.ucar.edu/datasets/ds083.2 and https://rda.ucar.edu/datasets/ds351.0. GDAS data is available at NOAA (National Oceanic and Atmospheric Administration) Air Resources Laboratory (ftp://arlftp.arlhq.noaa.gov/archives/gdas1). The simulation data used in this study are stored in the high performance computing center of Nanjing University and can be made available from the corresponding author upon request. Data processing techniques are available on request from the corresponding author. The source code of WRF-Chem model is archived on UCAR data repository (http://www2.mmm.ucar.edu/wrf/users/download). Lagrangian dispersion model can be acquired from the NOAA Air Resources Laboratory for the provision of the HYSPLIT (Hybrid Single-Particle Lagrangian Integrated Trajectory model) transport and dispersion model (http://www.ready.noaa.gov). The EEMD analysis code embedded in NCAR Command Language version 6.40 is available at https://www.earthsystemgrid.org/dataset/ncl.640.html.

## Supplementary Material

nwaa137_Supplement_File
