# Supplementary material for: Enhanced secondary pollution offset reduction of primary emissions during COVID-19 lockdown in China
Source: Natl Sci Rev. 2020 Jun 18;8(2):nwaa137. doi: 10.1093/nsr/nwaa137 (PMC7337733; doi:10.1093/nsr/nwaa137)
Supplement: nwaa137_Supplement_File [file nwaa137_supplement_file.docx]

**Supplementary Materials for**

**Enhanced secondary pollution offset reduction of primary emissions during COVID-19 lockdown in China**

Xin Huang^1*^, Aijun Ding^1*^, Jian Gao^2*^, Bo Zheng^3,4*^, Derong Zhou^1^, Ximeng Qi^1^, Rong Tang^1^, Jiaping Wang^1^, Chuanhua Ren^1^, Wei Nie^1^, Xuguang Chi^1^, Zheng Xu^1^, Liangduo Chen^1^, Yuanyuan Li^1^, Fei Che^2^, Nini Pang^2^, Haikun Wang^1^, Dan Tong^3,5^, Wei Qin^6^, Wei Cheng^6^, Weijing Liu^7^, Qingyan Fu^8^, Baoxian Liu^9^, Fahe Chai^2^, Steven J. Davis^3,5^, Qiang Zhang^3^, Kebin He^3,4^

^1^School of Atmospheric Sciences, Nanjing University, Nanjing 210023, China

^2^Chinese Research Academy of Environmental Sciences, Beijing 100012, China

^3^Department of Earth System Science, Tsinghua University, Beijing 100084, China;

^4^State Key Joint Laboratory of Environment Simulation and Pollution Control, School of Environment, Tsinghua University, Beijing 100084, China

^5^Department of Earth System Science, University of California, Irvine, CA 92697, USA.

^6^Jiangsu Environmental Monitoring Center, Nanjing, 210036, China.

^7^Jiangsu Provincial Academy of Environment Science, Nanjing 210036, China.

^8^Shanghai Environmental Monitoring Center, Shanghai 200030, China;

^9^Beijing Key Laboratory of Airborne Particulate Matter Monitoring Technology, Beijing Municipal Environmental Monitoring Center, Beijing 100048, China.

*These authors contributed equally to this work. Correspondence and requests for materials should be addressed to A. D. ([dingaj@nju.edu.cn](mailto:dingaj@nju.edu.cn)) or Q. Z. ([qiangzhang@tsinghua.edu.cn](mailto:qiangzhang@tsinghua.edu.cn)).

**This document includes Supplementary text, Supplementary Figures 1-14 and Supplementary Table 1.Supplementary text**

**Observational datasets and analysis method**

Measurements on hourly concentrations of air pollutants are analyzed to understand air quality change due to the most extreme COVID-19 lockdown in China. Concentration of NO_2_, O_3_, CO, SO_2_, PM_2.5_ and PM_10_ at more than 1500 stations are recorded hourly mainly based on Thermo Scientific samplers and analyzers. The geographic locations of these stations are marked in Fig. 1 (a-d). All these ground-based observations are archived at air monitoring data center of Ministry of Ecology and Environment of the People’s Republic of China (http://datacenter.mep.gov.cn), which are collected to derive the spatiotemporal variations in this study. As aforementioned, we divided the first two months of 2020 into two distinct time period according to the sharp drop of transportation activities due to the hit of COVID-19. The concentrations of multiple air pollutants during Pre-COVID and COVID-lock period are comprehensively compared. To clearly demonstrate the air quality variations, ensemble empirical mode decomposition (EEMD) is applied to decompose observational data and to derive the low frequency signals (timescale larger than 2 days) in Fig. 1e.

In addition, hourly PM_2.5_ chemical compositions are recorded at more than 40 air pollution observation stations in developed provinces and cities in eastern China. In Nanjing, besides routine measurements on PM_2.5_ concentration and its chemical compositions, we also conducted observations on particle size distribution, VOCs and other trace gases at the Stations for Observing Regional Processes of the Earth System (SORPES). The SORPES station is a cross-disciplinary research and experiment platform that was established in 2011 to understand the impact of human activities in the rapidly urbanized and industrialized eastern China region^[1]^. A scanning mobility particle sizer (SMPS), Aerodyne Soot Particle Aerosol Mass Spectrometer (SP-AMS), Time-of-Flight Aerosol Chemical Speciation Monitor (TOF-ACSM), compact IONICON PTR-TOF-MS trace VOC analyzer, and Aerodyne Research Long-Tof-CIMS were used to measure aerosol size distribution, fine particle chemical compositions, VOCs speciation, and sulfuric acid, respectively, at the station.

TROPOspheric Monitoring Instrument (TROPOMI) on board the Copernicus Sentinel-5 Precursor satellite provides retrievals of NO_2_ column amount^[2]^, which are also employed to illustrate the spatial pattern and temporal variation of air pollution around COVID-19 in China. The retrieved column burden is then averaged and compared in different regions for Pre-COVID and COVID-lock period.

**Emission reduction estimation due to the lockdown control**

Emission reduction is estimated using the bottom-up inventory model of Multi-resolution Emission Inventory for China (MEIC), developed by Tsinghua University^[3]^. To estimate emission reductions due to COVID-19 lockdown, we update China’s emissions data to Jan and Feb 2020 based on dynamic economic and industrial activity levels. The thermal power generation in the first two months of 2020 was 8.9% lower than that in 2019, while China generated 1.7% more thermal power in January and February 2019 than in 2018 (National Bureau of Statistics, http://www.stats.gov.cn). We then assume that the difference in the growth rates between 2019 and 2020 was the influence of COVID-19 lockdown on the power sector emissions. The same approach is applied to the industrial sector. For example, cement production in January and February 2020 was 29.5% lower than that in 2019, while China produced 0.5% more cement in January and February 2019 than in 2018. Cement emissions were then estimated to be reduced by 30% due to COVID-19. For the residential sector, emissions from the commercial use of boilers and stoves in the urban region were eliminated since the lockdown measure implemented, while emissions from residential heating and cooking in both urban and rural areas were assumed not affected. For the transport sector, decline in national traffic volume was estimated at 70% during the COVID-19 lockdown, according to the transportation index data. Activity level of off-road equipment such as construction machines was assumed approaching zero during the COVID-19 lockdown. As the result, on-road emissions declined by 70%, and the off-road emissions became zero during the lockdown. The detailed estimation of provincial emission reduction ratio of main trace gases and primary PM are presented in Table S1.


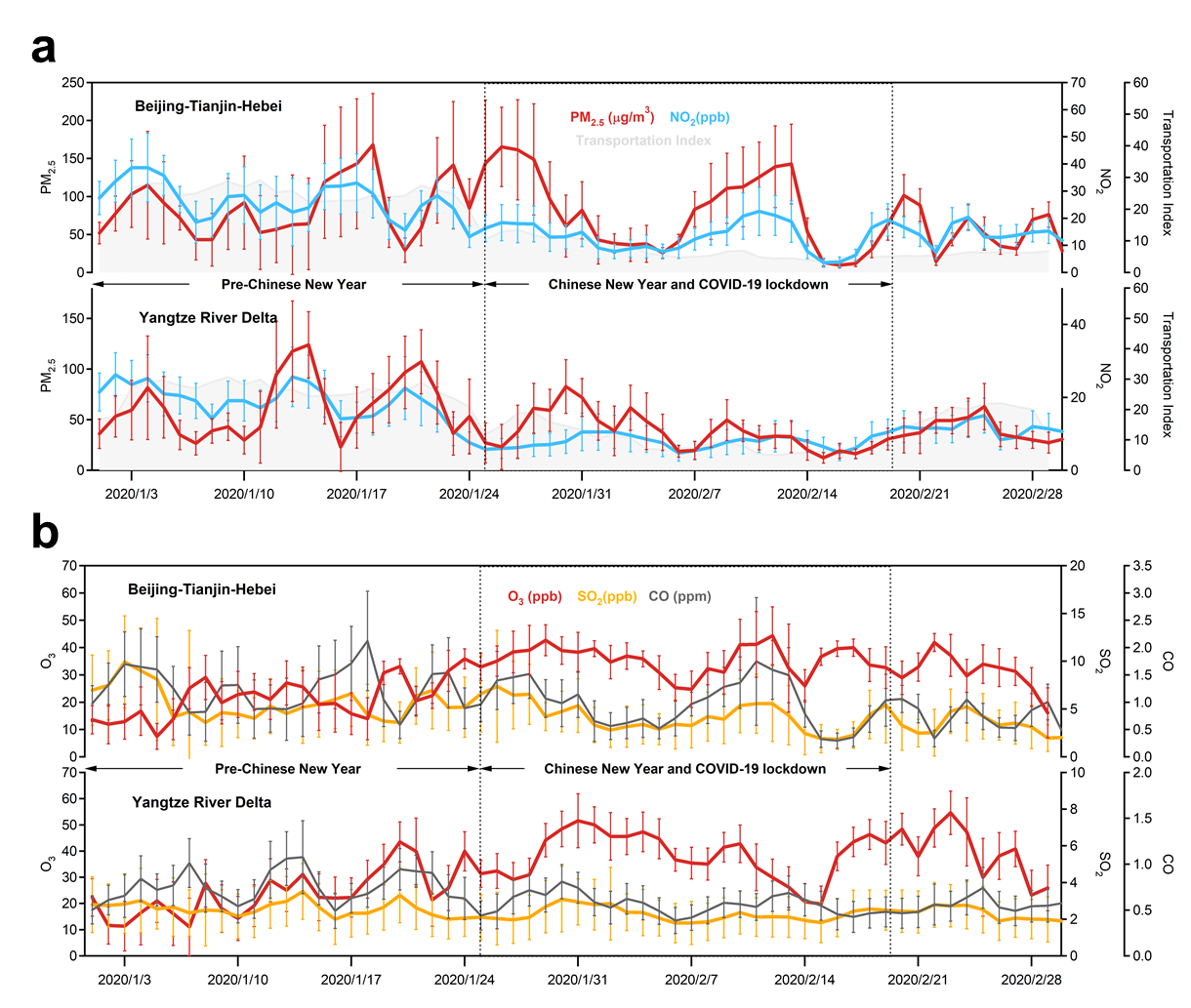


**Figure S1. Air quality before and during the COVID-19 pandemic in eastern China. a,** Time series of daily PM_2.5_, NO_2_ and transportation index in the BTH (upper panel) and the YRD (lower panel) during January-February 2020. **b,** Time series of daily O_3_, SO_2_, and CO in the BTH (upper panel) and the YRD (lower panel) during January-February 2020. Note that the solid lines are the average values and the error bars mark the standard deviations.


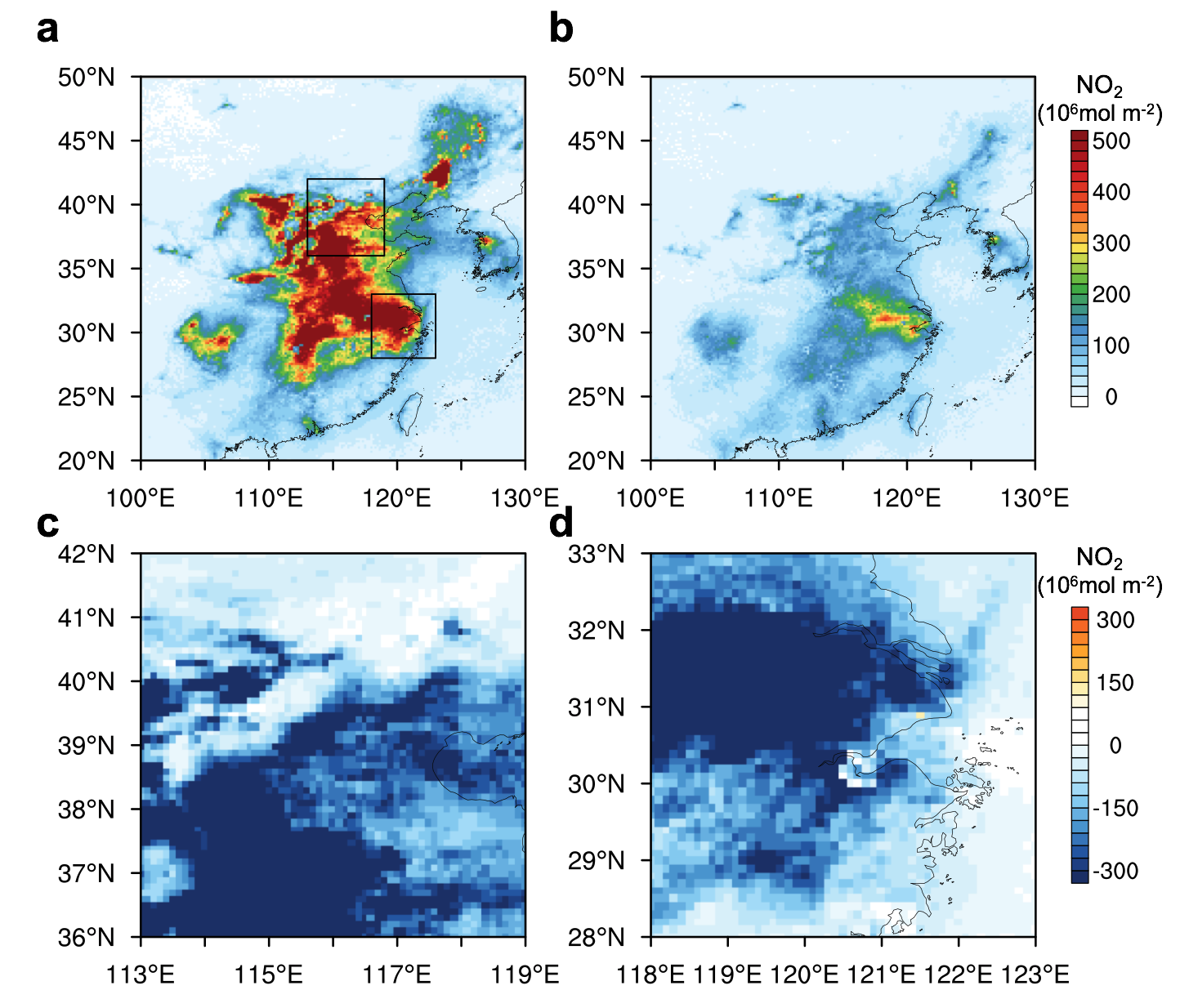


**Figure S2. Satellite retrievals of NO_2_ in eastern China before and during the COVID-19 pandemic. a-b,** Spatial distribution of averaged Trop-OMI tropospheric NO_2_ column in eastern China during the pre-COVID and COVID-lock periods. **c-d,** changes in Trop-OMI tropospheric NO_2_ column in the BTH and YRD regions.


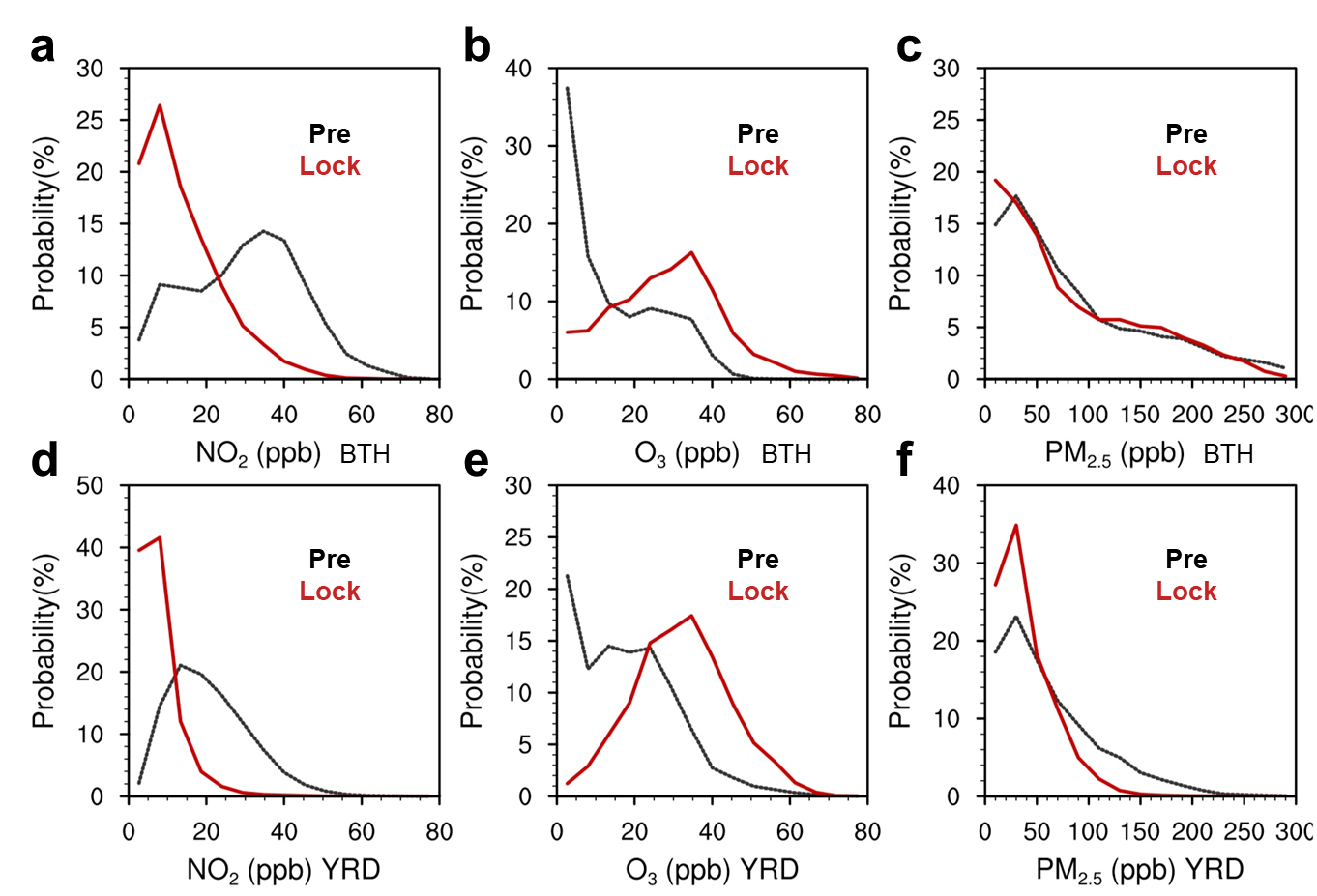


**Figure S3. Comparison of frequency distribution of air pollutants in eastern China due to COVID-19 lockdown. a-c,** Normalized frequency distributions of NO_2_, O_3_, and PM_2.5_ in the BTH region during the COVID-lock (red lines) and pre-COVID (black lines) periods. **d-f,** Normalized frequency distributions of NO_2_, O_3_, and PM_2.5_ in the YRD region.


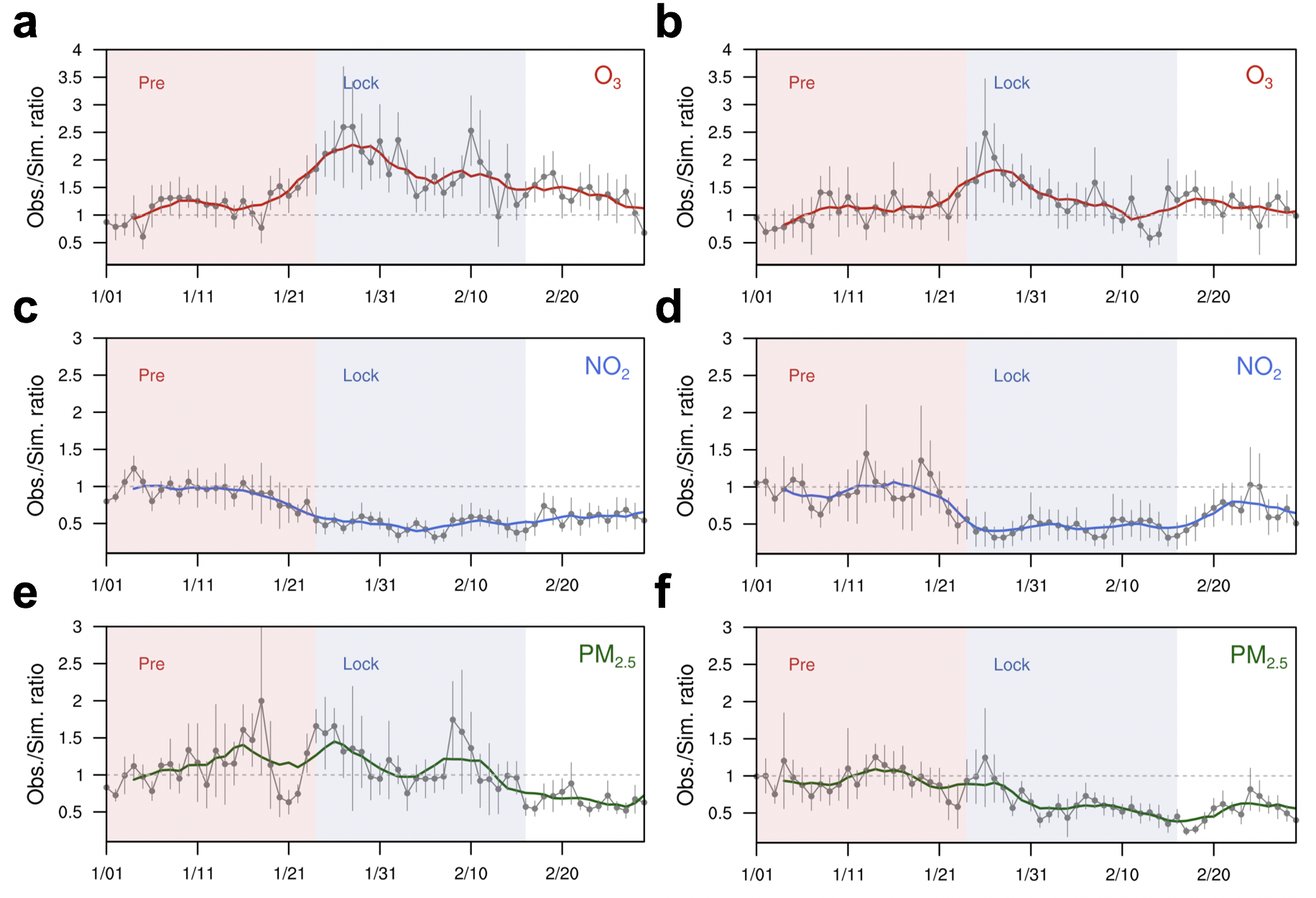


**Figure S4. Changes in air pollution indicated by comparison between observation and simulations. a, b,** Time series of daily ratio of observation verse WRF-Chem simulated O_3_ with fixed emission inventory in the BTH and YRD region. **c,d,** same as **a** and **b** but for NO_2_. **e.f,** same as **a** & **b** but for PM_2.5_. Note that the grey dots are the daily average values and the error bars mark the standard deviations. The red, blue and green lines present 7-day moving average for O_3_, NO_2_ and PM_2.5_.


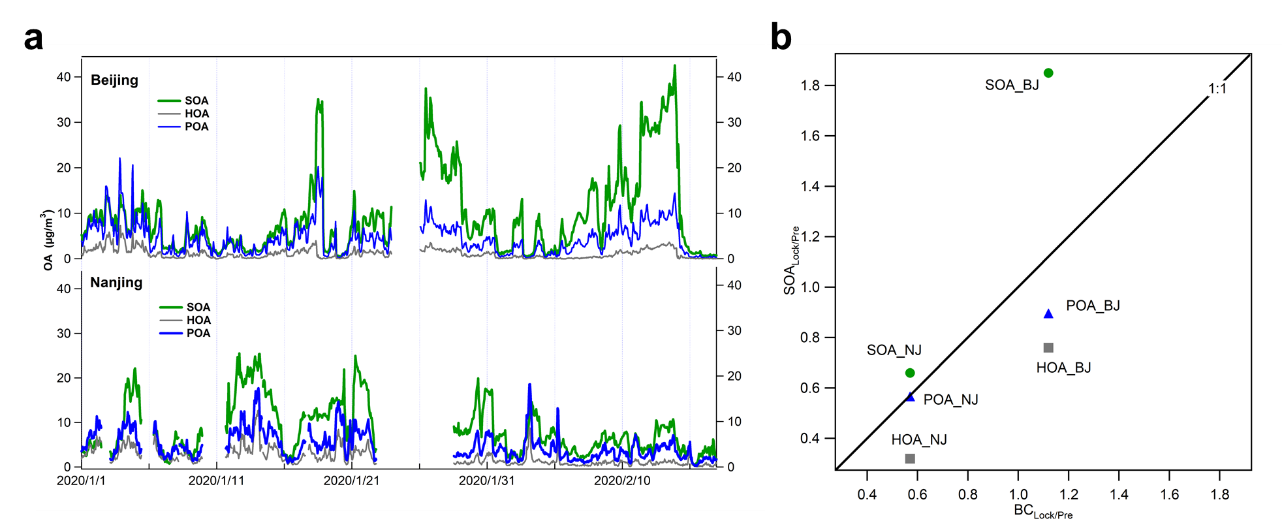


**Figure S5. Observation of organic aerosols in Nanjing and Beijing. a,** Time series of hydrocarbon-like organic aerosol (HOA), primary organic aerosol (POA) and secondary organic aerosol (SOA) before and after COVID-19 lock know in Beijing and Nanjing. **b**, Scatter plots of the ratios of SOA, POA and HOA as a function of the ratio of BC during the two periods. Note: Organic aerosol was measured using aerosol mass spectrometers (TOF-ACSM, Aerodyne) at both sites. Positive matrix factorization (PMF) was used to separate the measured OA to several factors, including HOA, COA, BBOA, LO-OOA and MO-OOA in Beijing, and HOA, BBOA, LO-OOA and MO-OOA in Nanjing. POA represented the sum of HOA, COA (if have) and BBOA, while SOA represented the sum of LO-OOA and MO-OOA.

**
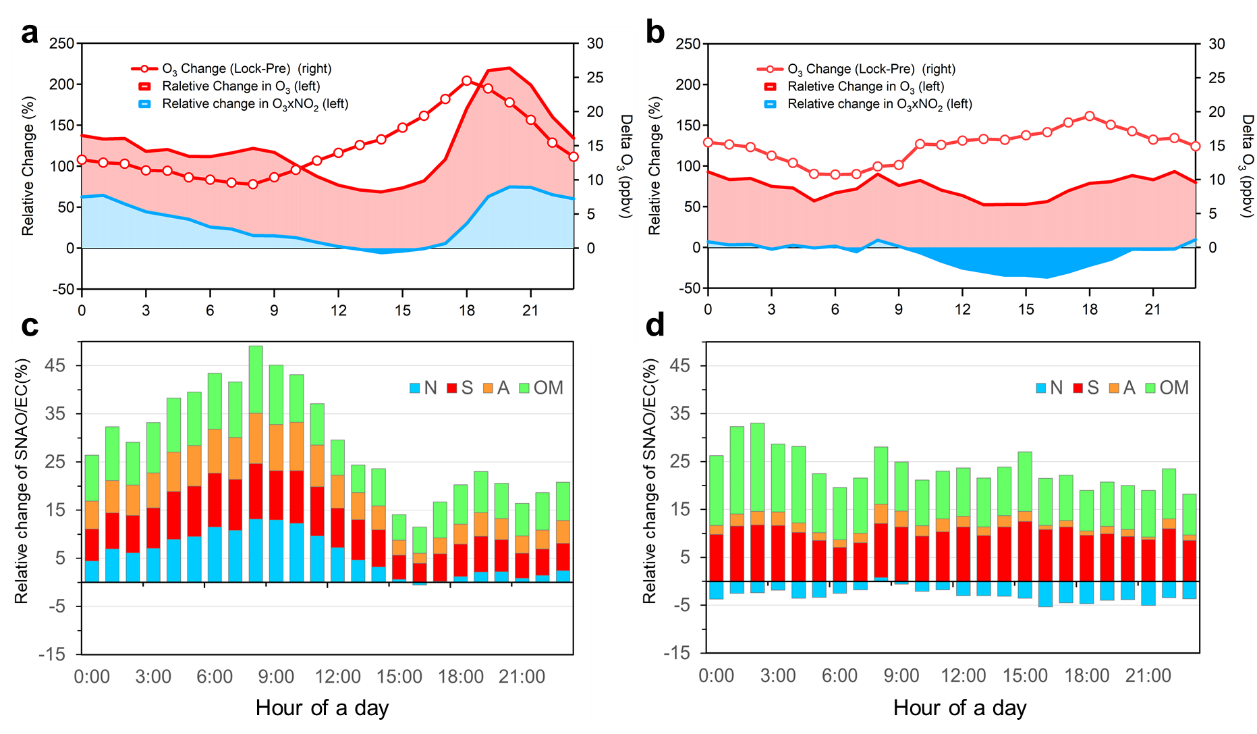
**

**Figure S6. Diurnal cycle of relative changes in secondary verse primary PM_2.5_ ratio, O_3_ and NO_3_ proxy in BTH and YRD. a, b,** Relative changes in O_3_ and O_3_*NO_2_ proxy between the COVID-lock and pre-COVID periods in BTH and YRD. **c, d,** Relative changes in SNAO/EC ratio weighted by the percentage of each compositions in PM_2.5_ between the COVID-lock and pre-COVID periods in BTH and YRD, respectively. Note: N, S, A and OM means nitrate, sulfate, ammonium and organic matter, respectively.


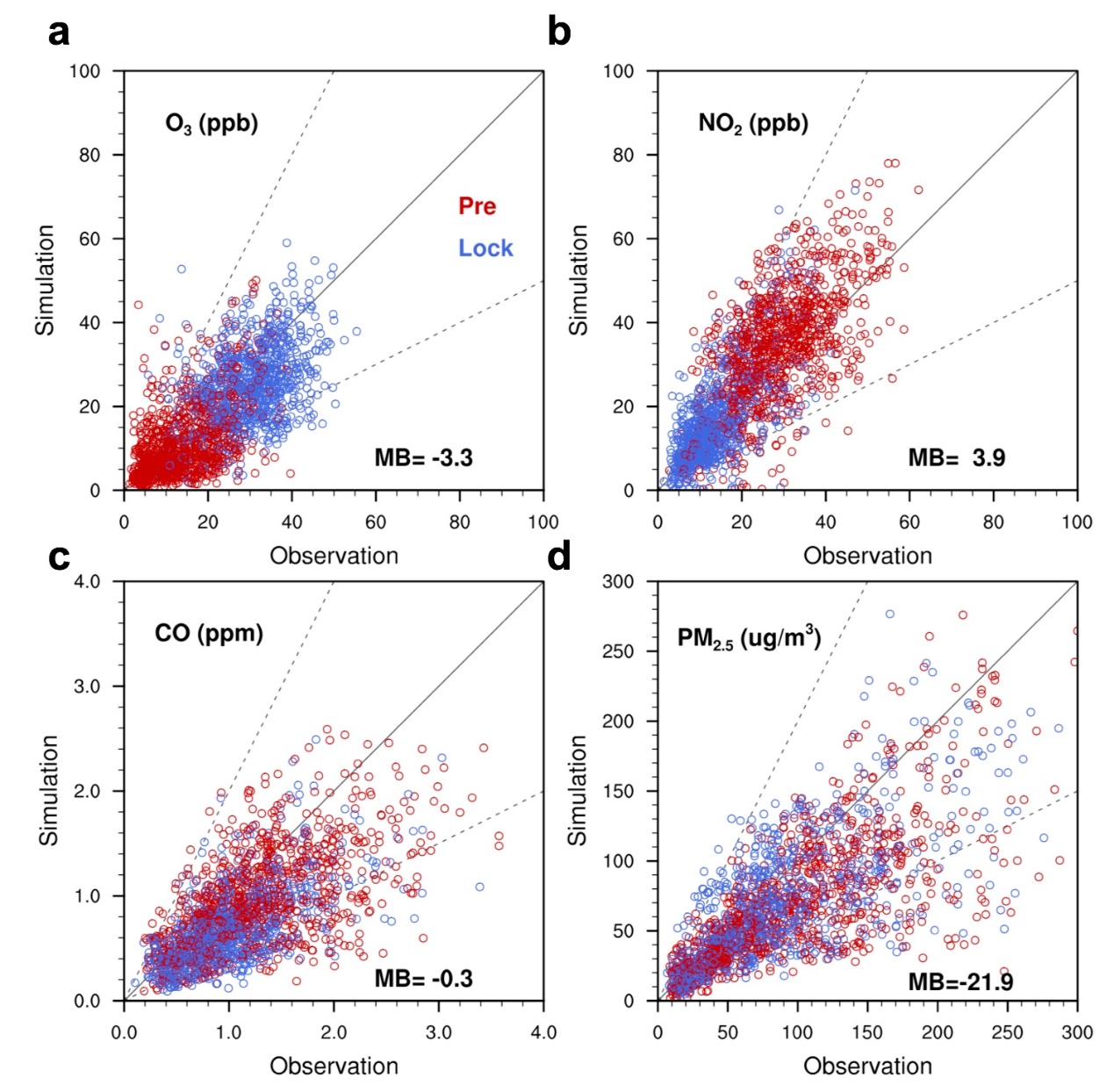


**Figure S7. Evaluation of WRF-Chem simulations with ambient measurements by the national air quality monitoring networks. a-d,** Scatter plots of simulation verse observation for O_3_, NO_2_, CO and PM_2.5_. Note that the red and blue markers present simulations and observations during the pre-COVID and COVID-lock period, respectively. Mean bias of modeled concentrations are labeled in the bottom right corner of each subplot.


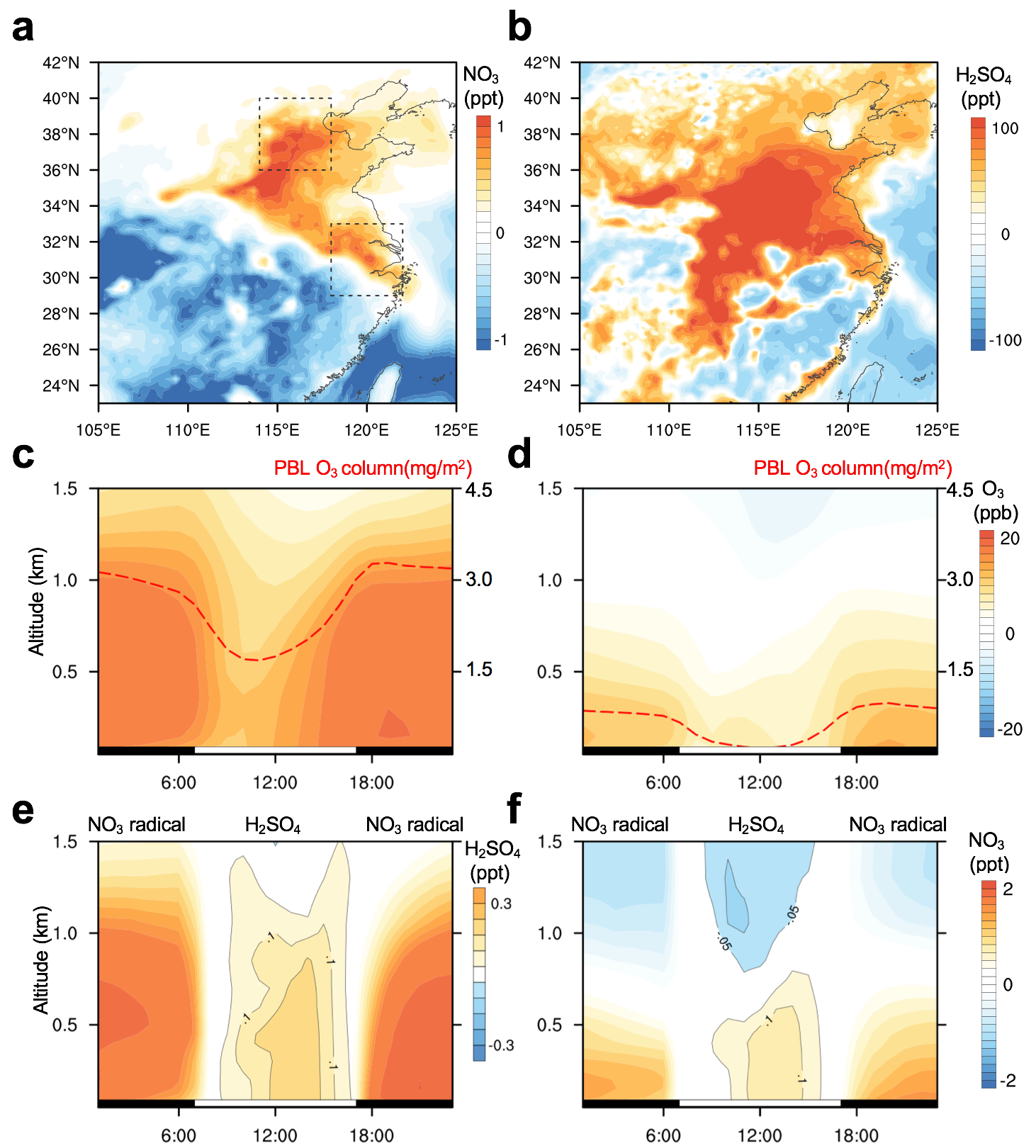


**Figure S8. Modeling evidences of enhanced oxidizing capacity during the COVID-19 lockdown period in BTH and YRD, China. a-b,** Spatial distribution of averaged changes in NO_3_ radical and H_2_SO_4_ between the COVID-lock and pre-COVID periods (Lock minus Pre) in BTH and YRD (dashed rectangles in **a**), China. **c, d**, Diurnal cycle of averaged vertical distribution of O_3_ and boundary layer (0-1 km) O_3_ column between the two period in BTH and YRD. **e, f,** Diurnal cycle of NO_3_ radical and H_2_SO_4_ between the two period in BTH and YRD. Note: The black and white bars near the x axis in **c-f** indicate the nighttime and daytime of a day.


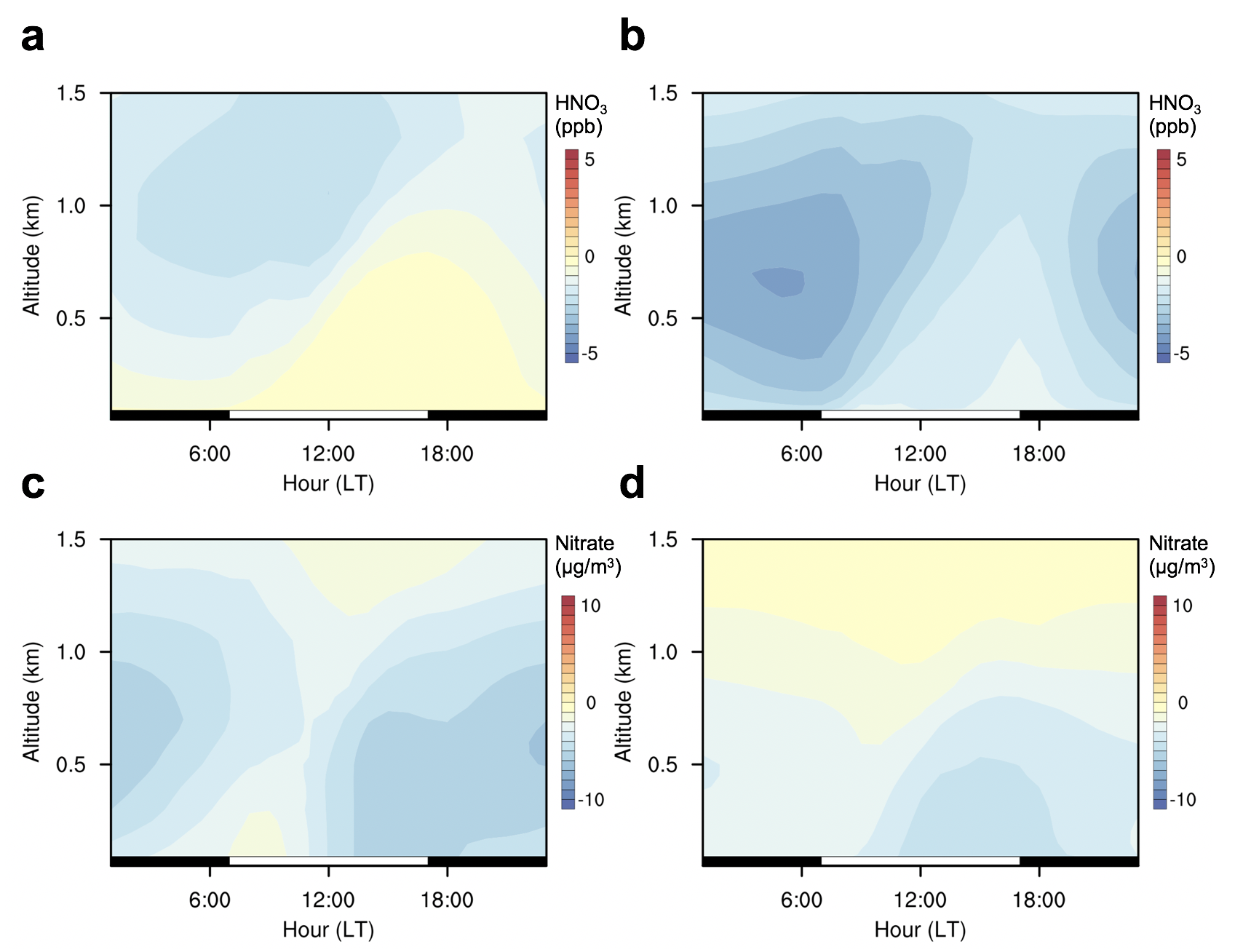


**Figure S9. Comparison of simulated diurnal cycles of vertical distribution of HNO_3_ and nitrate in BTH and YRD. a, b,** WRF-Chem simulated changes in the diurnal cycle of HNO_3_ vertical distribution in BTH and YRD. **c,d,** same as **a** and **b** but for nitrate. The black and white bars near the x axis indicate the nighttime and daytime of a day.


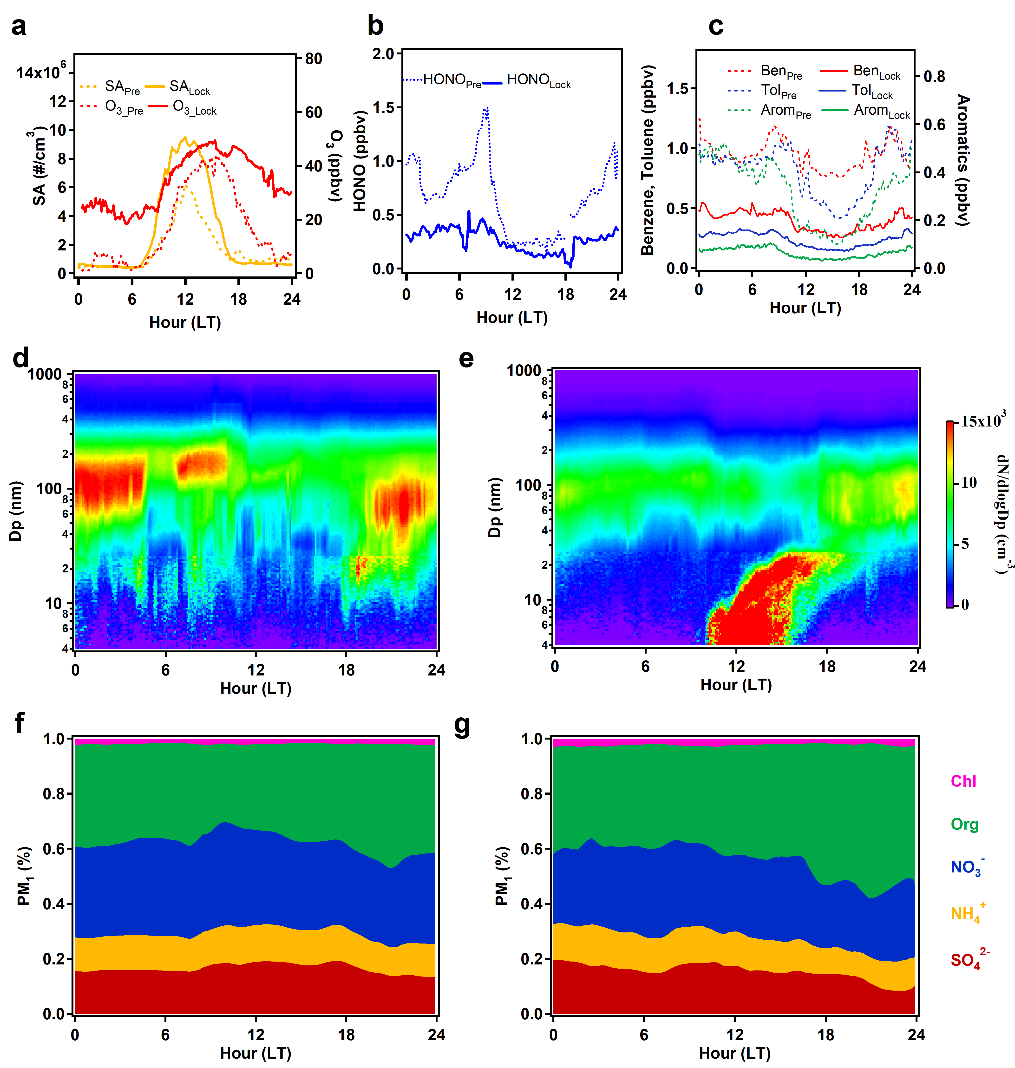


**Figure 10. Observations of trace gases and aerosols at the SORPES station of Nanjing during the pre-COVID and COVID-lock periods. a-c,** Diurnal cycle of O_3_ and sulfuric acid (SA), HONO, and VOCs species in sunny days during the two periods. **d-e,** Diurnal cycle of aerosol size during pre-COVID and COVID-lock periods. **f-g,** Diurnal cycles of percentage of PM_1_ speciation the pre-COVID and COVID-lock periods. Note: Ben- Benzene, Tol-Toluene, Arom- C8-C10 aromatics. During the pre-COVID period there is 0 day with NPF, but 10 days (71.4%) out of 14 sunny days featured clear NPF events during the COVID-lock period.


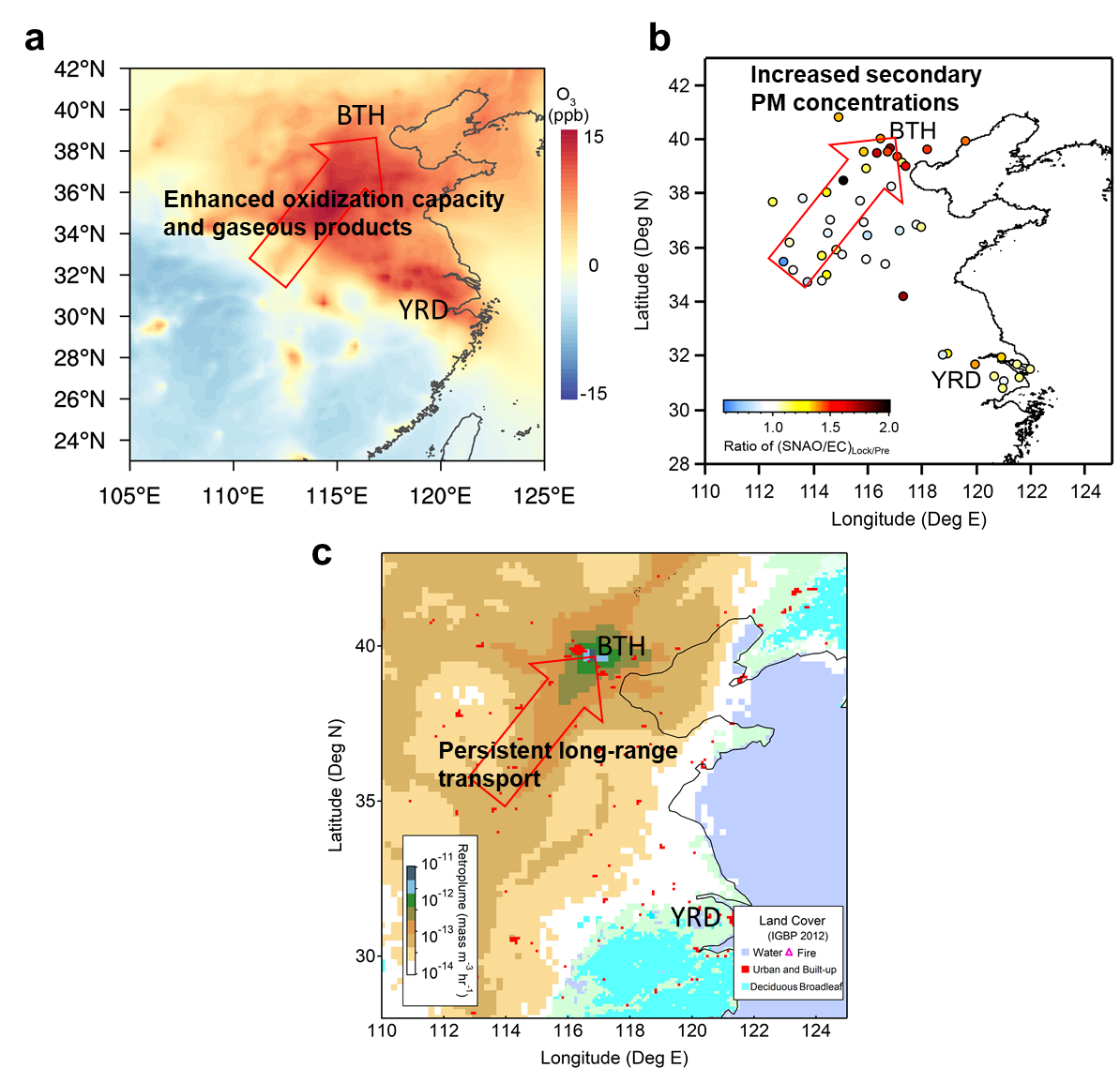


**Figure S11. Linkage between enhanced oxidizing capacity and gaseous oxidation products, aerosol concentrations and long-range transport induced by meteorology for the BTH region during the COVID-19 lockdown. a,** Same as Fig. 3a. **b**, Same as Fig. 2a. **c**, Averaged retroplume for air masses arrived at Beijing during the COVID-lock period. Note: The retroplume in **c** was calculated using Lagrangian dispersion model HYSPLIT backwardly for a 7-day period for every 3 hours during the lockdown period.


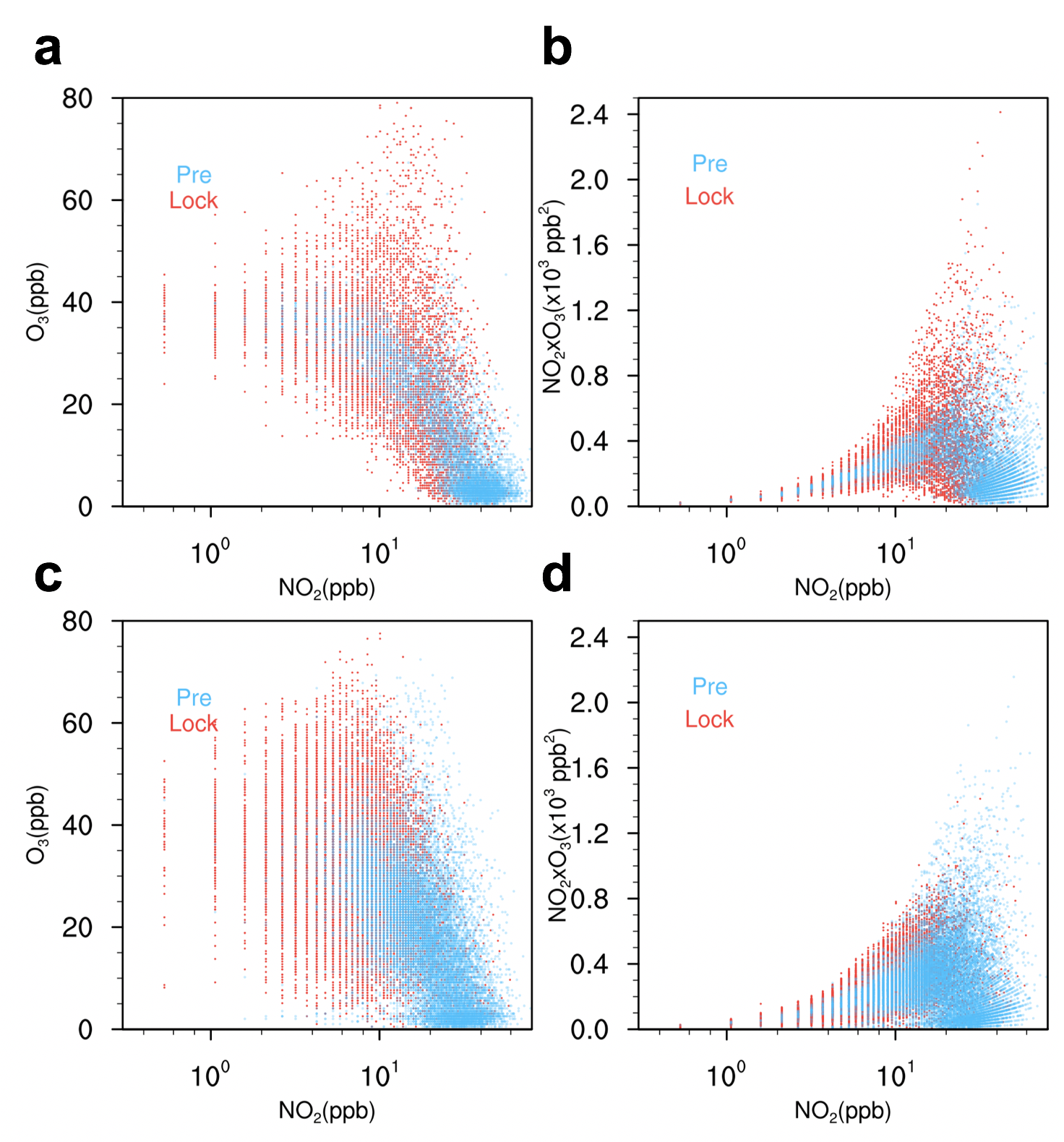


**Figure S12. Observed non-linear relationship between main oxidants and NO_2_ caused by the COVID-19 lockdown in China. a, b,** Scatter plots of O_3_ and proxy of NO_3_ radical (NO_2_*O_3_) versus NO_2_ between the COVID-lock and pre-COVID periods in BTH of China. **c, d,** Same as **a** and **b** but for the YRD region.
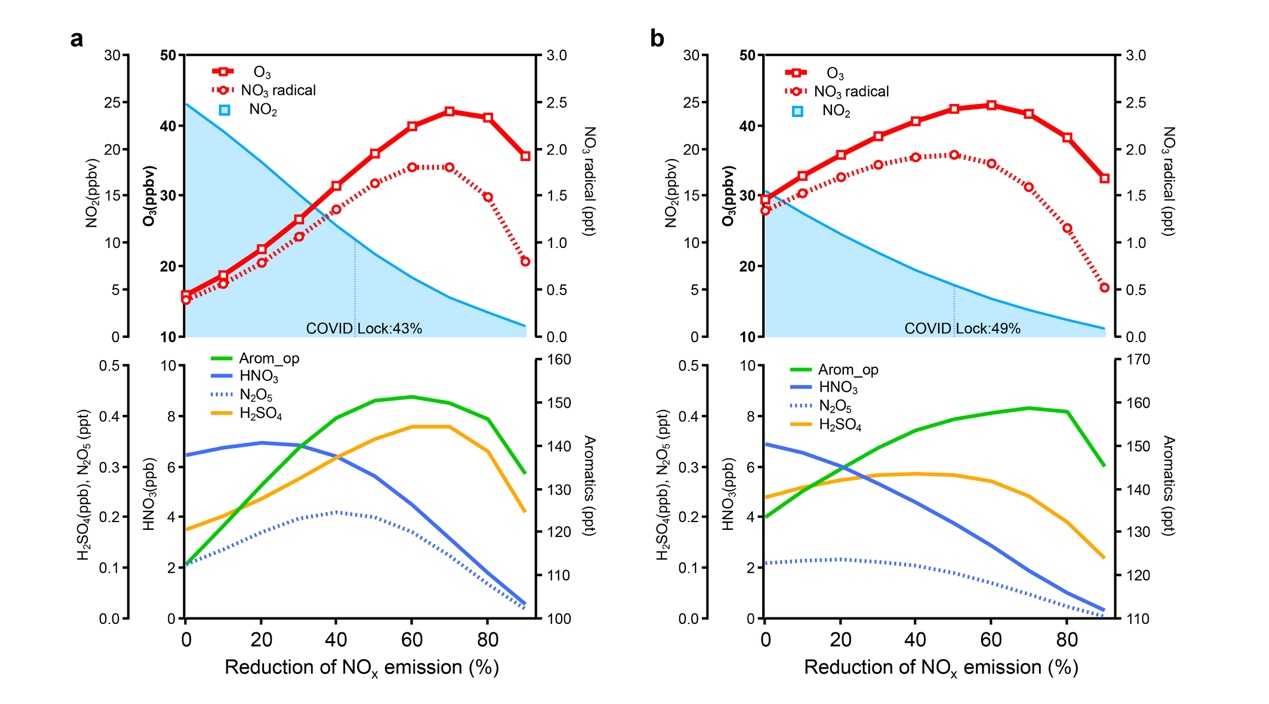


**Figure S13. Response of atmospheric oxidizing capacity and gaseous oxidation products to NO_x_ emission reduction. a, b,** Changes of NO_2_, O_3_, and NO_3_ radical (upper panel) and gaseous oxidation products (lower panel) as a function of emission reduction in the BTH and YRD regions, respectively. Note: BTH and YRD are defined as the domains of (36°N-40°N, 114°E-118°E) and (29°N-33°N, 118°E-122°E). Arom_op means oxidization products of aromatics. The averaged emission reduction of NO_x_ for the two regions are marked in the upper panel.


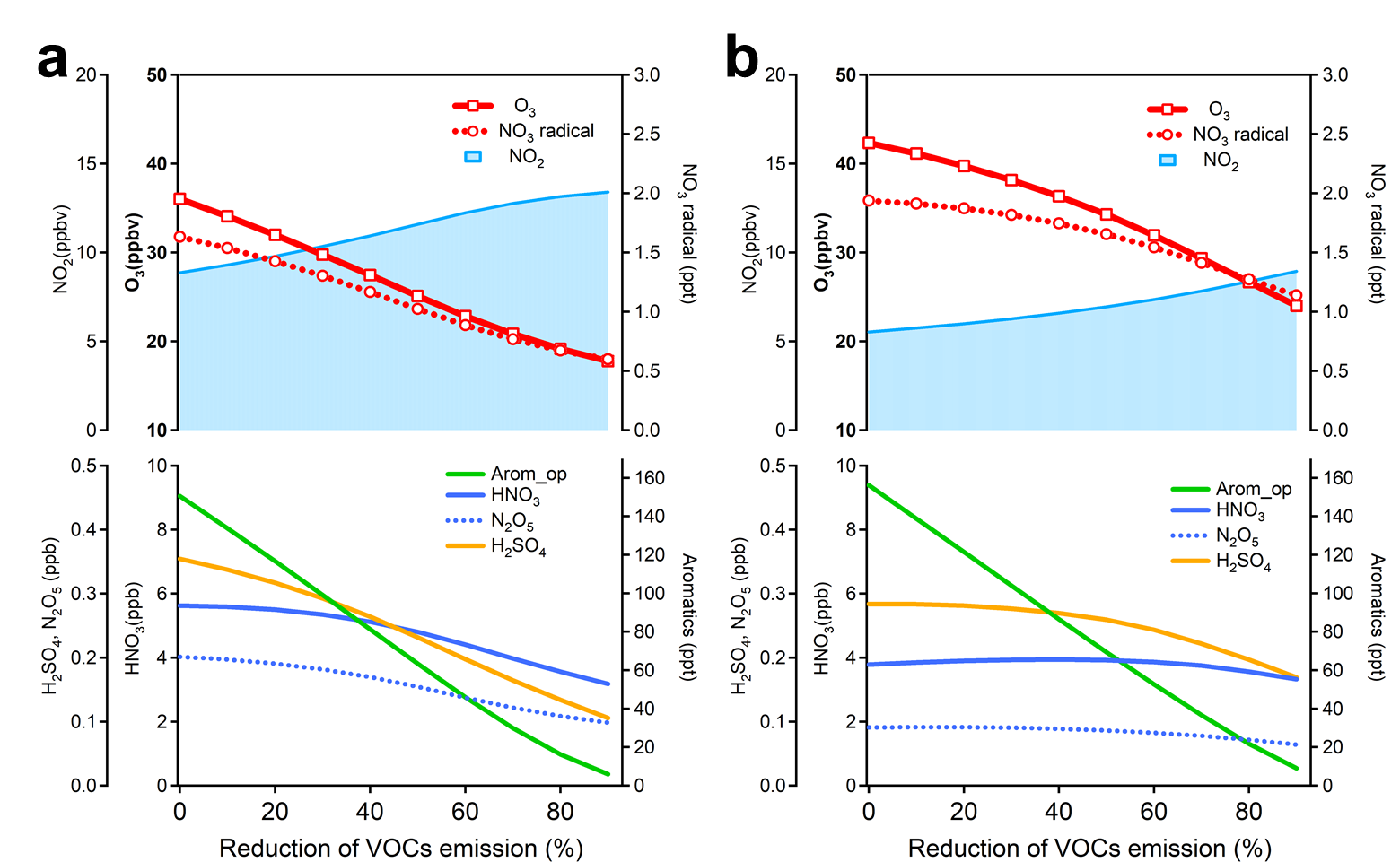


**Figure S14. Response of atmospheric oxidizing capacity to VOCs emission reductions in the BTH and YRD, China. a, b,** Changes of NO_2_, O_3_, and NO_3_ radical (upper panel) and gaseous products (proxy) (lower panel) as a function of emission reductions (NO_x_: 50%, VOCs: 10-90%) in the BTH and YRD regions, respectively. Note: BTH and YRD are defined as the domains of (36°N-40°N, 114°E-118°E) and (29°N-33°N, 118°E-122°E). Arom_op means oxidization products of aromatics.

**Table S1. Estimation of provincial emission reduction ratio (%) of CO, NO_x_, SO_2_, VOC, PM_2.5_, BC, OC due to COVID-19 lockdown in China.**

| **Province** | **CO** | **NO_x_** | **SO_2_** | **VOC** | **PM_2.5_** | **BC** | **OC** |
| --- | --- | --- | --- | --- | --- | --- | --- |
| Beijing | 22% | 45% | 26% | 45% | 18% | 46% | 8% |
| Tianjin | 21% | 38% | 20% | 41% | 14% | 22% | 6% |
| Hebei | 15% | 45% | 16% | 36% | 12% | 17% | 5% |
| Shanxi | 18% | 40% | 20% | 33% | 16% | 19% | 10% |
| Inner Mongolia | 14% | 29% | 15% | 34% | 13% | 16% | 6% |
| Liaoning | 21% | 40% | 28% | 36% | 16% | 28% | 8% |
| Jilin | 16% | 39% | 23% | 34% | 13% | 18% | 5% |
| Heilongjiang | 17% | 37% | 27% | 28% | 13% | 15% | 7% |
| Shanghai | 35% | 48% | 42% | 45% | 34% | 54% | 42% |
| Jiangsu | 23% | 50% | 26% | 41% | 16% | 35% | 7% |
| Zhejiang | 41% | 50% | 29% | 45% | 30% | 49% | 20% |
| Anhui | 14% | 56% | 22% | 31% | 11% | 22% | 4% |
| Fujian | 29% | 51% | 30% | 42% | 19% | 31% | 7% |
| Jiangxi | 24% | 53% | 21% | 43% | 19% | 30% | 9% |
| Shandong | 23% | 50% | 25% | 39% | 19% | 35% | 9% |
| Henan | 23% | 57% | 22% | 41% | 18% | 35% | 8% |
| Hubei | 19% | 55% | 23% | 35% | 16% | 23% | 10% |
| Hunan | 22% | 51% | 25% | 36% | 20% | 24% | 15% |
| Guangdong | 38% | 50% | 33% | 46% | 27% | 42% | 13% |
| Guangxi | 24% | 50% | 28% | 39% | 17% | 27% | 5% |
| Hainan | 24% | 44% | 25% | 36% | 14% | 25% | 4% |
| Chongqing | 18% | 53% | 32% | 37% | 14% | 20% | 4% |
| Sichuan | 16% | 50% | 27% | 33% | 9% | 15% | 3% |
| Guizhou | 24% | 39% | 25% | 30% | 22% | 25% | 20% |
| Yunnan | 24% | 51% | 25% | 41% | 18% | 21% | 8% |
| Tibet | 16% | 35% | 15% | 35% | 14% | 14% | 5% |
| Shaanxi | 19% | 45% | 18% | 34% | 13% | 22% | 5% |
| Gansu | 13% | 47% | 16% | 29% | 9% | 13% | 3% |
| Qinghai | 23% | 46% | 22% | 39% | 20% | 20% | 7% |
| Ningxia | 24% | 36% | 24% | 39% | 20% | 23% | 8% |
| Xinjiang | 16% | 35% | 15% | 35% | 14% | 14% | 5% |

**References**

1 Ding A J *et al.* Significant reduction of PM2.5 in eastern China due to regional-scale emission control: evidence from SORPES in 2011-2018. *Atmos Chem Phys* 2019; **19**: 11791-11801.

2 Veefkind J *et al.* TROPOMI on the ESA Sentinel-5 Precursor: A GMES mission for global observations of the atmospheric composition for climate, air quality and ozone layer applications. *Remote Sens Environ* 2012; **120**: 70-83.

3 Li M *et al.* Mapping Asian anthropogenic emissions of non-methane volatile organic compounds to multiple chemical mechanisms. *Atmos Chem Phys* 2014; **14**: 5617-5638.
